# Facilitators and Barriers to a Hospital-Based Communication Skills Training Programme: An Interview Study

**DOI:** 10.3390/ijerph20064834

**Published:** 2023-03-09

**Authors:** Maiken Wolderslund, Karin Waidtløw, Poul-Erik Kofoed, Jette Ammentorp

**Affiliations:** 1 Centre for Research in Patient Communication, Odense University Hospital, 5000 Odense, Denmarkjette.ammentorp@rsyd.dk (J.A.); 2 Department of Clinical Research, University of Southern Denmark, 5000 Odense, Denmark; 3 Department of Paediatrics and Adolescent Medicine, Lillebaelt Hospital, University Hospital of Southern Denmark, 6000 Kolding, Denmark; 4 Department of Regional Health Research, University of Southern Denmark, 5000 Odense, Denmark

**Keywords:** health communication, patient-centred care, staff development, clinical competence, provider–patient relations, program evaluation, quality of care

## Abstract

This study aimed to investigate the facilitators and barriers experienced by the department management (DMs) and communication skills trainers (trainers) during the implementation of a 3-day communication skills training (CST) programme for healthcare professionals (HCPs). Thus, we conducted semi-structured interviews with 23 DMs and 10 trainers from 11 departments concurrently implementing the CST programme. Thematic analysis was undertaken to elucidate the themes across the interviews. Five themes were developed: resource consumption; obstacles; management support; efforts and outcomes; and a lack of systematic follow-up. Although the DMs and trainers were largely in agreement, the theme of a lack of systematic follow-up was derived exclusively from the trainers, as were two of the subthemes within obstacles: (b) seniority, profession, and cultural differences, and (c) the trainers’ competencies. The greatest perceived barrier was resource consumption. In addition, DMs found planning and staff resistance to be a challenge. However, the HCPs’ resistance diminished or even changed to satisfaction after participating. The mandatory approach served as both a facilitator and a barrier; DMs’ support was an essential facilitator. Explicit communication related to resource demands, planning, and participation is crucial, as is management support and the allocation of resources.

## 1. Introduction

Systematic postgraduate communication skills training (CST) is rarely implemented, despite evidence that communication skills at a sufficient level are essential for healthcare professionals’ (HCPs) ability to deliver patient-centred care (PCC) [1,2]. Furthermore, HCPs often experience challenges in integrating patients’ perspectives and preferences in care [3,4,5,6,7,8]. Ignoring the importance of the continuous development of communication skills can result in poor communication, leave patients with inadequate care, and have potentially severe consequences for HCPs, resulting in stress and emotional burnout [9,10,11]. A recent study by Weiner et al. [12] supports this by showing that the ability to identify and address the patients’ context was associated with a greater likelihood of improved outcomes.

Various CST methods can be used to enhance HCPs’ communication skills encompassing PCC [13,14,15,16,17,18,19]. The efficient implementation and transfer of skills into daily practice are related both to the methods used [1,20], the complexity of the clinical skills required to conduct patient-centred communication [16,21,22,23,24,25,26], and organisational and individual issues and concerns [27]. Several facilitating factors and barriers can be identified; however, combining approaches is the most effective strategy to support the transfer of acquired skills to the workplace [28,29]. Growing awareness of the importance of developing and maintaining communicative skills has increased the interest in organisation-based interventions implementing CST for HCPs [30,31].

However, CST is rarely transferred into practice, and research on how to systematically implement and maintain the skills at an organisational level is limited [2,20,30,32]. A recent publication on a CST program for physicians providing advanced cancer care concluded that one should expect resistance and scepticism when implementing CST and thus highlights the importance of teaming up with management and gatekeepers and being prepared for implementation to take time [33]. The positive results in studies investigating the effect of CST on HCPs and patients conducted in Lillebaelt University Hospital [34,35,36,37,38] paved the way for an organisation-based implementation, including all HCPs at the hospital. The CST program ‘Clear-cut communication with patients’ includes (a) training of the trainers, (b) training of the staff, and (c) initiatives for the maintenance of communication skills [39,40].

This study aimed to investigate the department management’s (DMs) and communication skills trainers’ (trainers) experiences of implementing the 3-day multidisciplinary CST programme for all HCPs to identify facilitators and barriers to the process [38].

## 2. Materials and Methods

### 2.1. Design

An interview study with DMs and trainers investigating barriers and facilitators associated with implementing a CST programme.

### 2.2. Setting and Participants

Lillebaelt University Hospital is a Danish regional hospital with ten surgical, eight medical, and ten clinical-service departments. Due to the long process of implementing the CST programme, it was decided to start the evaluation process after eleven representative departments had been included: four surgical departments, four medical departments, and three clinical-service departments.

Across departments, 20 interviews were conducted with 23 DMs and ten trainers (Table 1).

### 2.3. Data Collection

A semi-structured interview guide was developed based on the Re-Aim framework, laying the foundation for the implementation process [40]. This framework provides a systematic way to evaluate an intervention’s potential for impact in real-world settings, given its ability to provide a comprehensive evaluation of an intervention’s effectiveness and potential for widespread dissemination and adoption, which is known to be important from earlier implementation research [41,42,43]. It included questions to elucidate the study participants’ experiences of the practical and logistic issues during the implementation of the CST programme, e.g., How has the course content been received in the department? Can you describe some of your experiences with the process in the department? If I take a trip through your department to get a response to the courses, the process and the results—what will I hear? Has it changed during the process? Have there been any specific managerial challenges?

Interviews were conducted from 2012 to 2015 and were audio-recorded. Due to the work schedule and busyness in the department, we chose individual interviews with the trainers, whereas the DMs (consisting of two or three managers) were interviewed together.

### 2.4. Communication Skills Training

The CST was based on the Calgary-Cambridge Guide [16] and conducted in groups of 8–10 persons using different experiential and didactic methods, including role-play, feedback, and group discussions. The training also included feedback from peers and trainers on a mandatory video recording of an actual patient conversation. At each department, 4–8 HCPs were trained to conduct the CST in their department [38]. Supplementary information on the content of the CST and the education of trainers can be found in previously published papers [39,40,44,45].

### 2.5. Implementation Process and Organisation

The hospital management and the DMs initially decided to implement CST as a mandatory programme. However, given the strong objection by a small group of around ten HCPs in the starting phase of the implementation, the HCPs were allowed to ask their leaders to be exempted. However, to our knowledge, only a few HCPs asked to be exempted throughout the implementation.

**Figure 1:**
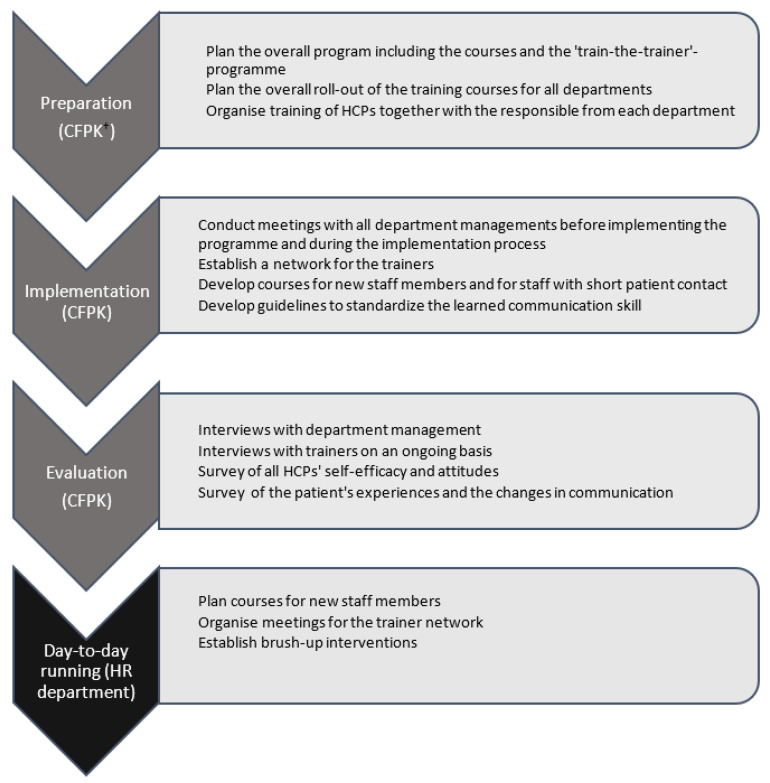
Overview of the entire implementation process. ^†^ CFPK: Centre for Research in Patient Communication, Odense, Denmark.

Our research unit, the Centre for Research in Patient Communication (CFPK), planned all aspects of the implementation process, including start-up meetings, follow-up, and evaluation (Figure 1).

### 2.6. Data Analysis

All 20 interviews were recorded and transferred to the NVivo 11 qualitative data analysis software. Regrettably, when reopening the audio files, seven were inaccessible for technical reasons; these comprised three interviews with DMs and four individual interviews with trainers. However, meaningful statements in the missing audio files had previously been transcribed, leaving us with 146 citations of value, thus included in the analysis. The remaining 13 interview audio files were carefully listened to twice, while the initial and supplemental notes were created simultaneously. During a third listening, meaningful statements were transcribed and subsequently coded based on the thematic analysis results, as described by Taylor and Bogdan [46]. Later, patterns were identified, and the main themes and subthemes were developed.

### 2.7. Ethics

The participating DMs and trainers provided oral consent before participation. The study was approved by the Danish Data Protection Agency but did not require approval by the National Committee on Health Research Ethics; however, the ethical principles of the Declaration of Helsinki were followed.

## 3. Results

Table 2 shows the five main themes and subthemes developed from the data analysis. The DMs and trainers mainly agreed; however, the subthemes b. seniority, profession, and cultural differences, and c. the trainers’ competencies, and the main theme, a lack of systematic follow-up, were exclusively derived from the interviews with trainers.

### 3.1. Resource Consumption

All DMs reported that the CST courses were very resource-demanding, given the allocation of working hours to allow HCPs to participate in the courses. Furthermore, all interviewees declared that planning had been comprehensive and exhausting and that it was not easy to maintain production, resulting in decreased planned clinical activity such as patient consultations and non-urgent operations. The main challenges were planning many courses within a tight schedule and rescheduling the employees’ work plans whenever unforeseen events occurred.

Furthermore, pre-course practical issues were considered a burden: singling out, encouraging, and motivating the most suited persons for positions as trainers, organising the distribution of preparation materials, and video-recording the communication situations in clinical practice. DMs and trainers suggested establishing a guideline for preparing the courses.

### 3.2. Obstacles

Different levels of dissatisfaction are to be expected when implementing an intervention such as this. Factors associated with this frustration surfaced during the interviews and will be described in detail in the following five subthemes.

#### 3.2.1. Teaching Methods

All participants reported that role-play and video recordings were their primary concerns. A DM stated, “I have seen nurses, old colleagues, crying and saying, ‘I’m not doing it!’”, and another, “I have had conversations with employees saying: ‘If I must attend, I will resign’”. The interviews elucidated that the main reason for the resistance may be fear of poor communication skills being revealed during the role-play and the video recordings from their clinical practice. One of the DMs expressed it as follows: “Maybe it’s the fear of being exposed, revealing you are not as good as you think you are”. However, having attended the CST, scepticism often changed, as elaborated in Section 3.2.5.

#### 3.2.2. Seniority, Profession, and Cultural Differences

According to the trainers, senior employees with many years of experience had, by large, difficulties acknowledging the necessity of CST: “How much novelty can there be in communicating? We have been doing this for 40 years” (a trainer, describing a typical reaction from senior HCPs). Another trainer explained, “It’s hard for them to appreciate that there might be things you could get better at”.

Besides seniority, the profession was decisive for the degree of resistance. Across specialities and departments, doctors demonstrated the most significant scepticism: “The doctors, especially the chief physicians, have had difficulties attending a course they had not chosen themselves. This was probably one of the biggest challenges” (reported by one of the trainers). As with the senior staff members, they were inclined to have the following attitude, as another trainer explained: “I’ve been communicating for years, so I know how to do that”. Doctors with a non-Danish cultural background expressed similar opinions; however, as indicated by the trainers, it could be due to other reasons: “They are used to being the most important. They are not used to everyone being equal and replaceable”.

The DMs were responsible for informing the staff at their departments about the CST. There were no common guidelines for how this introduction should proceed, and the process was not documented. As described, the Centre for Research in Patient Communication (CFPK) assisted in the planning of the implementation and helped to conduct start-up meetings. In some departments, resistance and negativity arose and spread among the HCPs, mainly due to the mandatory nature of the programme, resulting in the DMs having to intervene.

“The DM started showing up at the beginning of each course, explaining the background and the importance of the course. That worked”, as stated by a trainer.

#### 3.2.3. Trainers’ Competencies

The trainers were all HCPs with limited or no previous teaching experience. Some of them experienced that the participants questioned their competencies. One of the trainers said, “That has been the biggest problem, I think (...) there is an expectation of meeting an expert”. Even though the number of participants who questioned the trainers’ competencies was small, their opinions affected the trainers. Consequently, some felt insecure and nervous: “I have butterflies in my stomach every time I have to teach”, and “I am completely wasted when I go home after three days. I never know what awaits me”.

#### 3.2.4. Sense-Making

When the CST was instituted, it was compared with other mandatory hospital projects. A DM stated, “Exactly this type of project, which is being forced down over the departments from above, can be difficult to implement”.

According to the DMs and the trainers, the key to accommodating and changing the resistance lies in explaining the background and goal, thereby promoting meaningfulness and understanding. “The goal must be clear, not just presented as a must-do task” (DM). A trainer supported this: “If it does not make sense, nothing happens”.

#### 3.2.5. Transforming Reluctance by Doing

Despite the described resistance, reluctance and apprehension often faded and even changed to satisfaction after attending the CST: “Scepticism transformed after day one”, a trainer stated. Two DMs supported this: “An employee said at a staff meeting, ‘I went with the expectation simply to get it done with, but it was excellent. We gained something from it. It was not as transgressive as feared’”.

Although experiences from the CST varied from department to department, as well as at the individual level, the majority provided favourable feedback on the training: “I have not heard anyone say they did not think it was good. I have not. Everyone I have asked has been excited about it” (DM), and “They think it has helped and that it is useful” (trainer).

### 3.3. Management Support

Support from the hospital management and DMs played a significant role. It included providing the trainers with time off to prepare for the CST, handling resistance, and showing up at the beginning of the CST to discuss the importance of the training. Finally, most DMs attended the CST themselves.

The DMs were highly conscious of their roles: “It’s a mandatory project, but it’s important that we turn it into something positive. It’s essential that we back it up for it to become a success”.

Likewise, all trainers were aware of the importance of the support: “Our DM has taken the lead and has put it on the agenda”. In some cases, the DM interferences had been less appropriate, as stated by a trainer: “Our DM said, ‘If you want to be employed in this department, this course is something you must do’”. However, the trainers mainly described the support as positive.

### 3.4. Efforts and Outcomes

One topic included in the interview guide was the balance between the efforts invested in the intervention and the experienced outcomes. DMs and trainers highlighted the advantages of achieving a universal language through the CST: “This also gives us a common perception of communication and a common language” (DM). The trainers indicated that the employees used the communication skills and the structure for the consultation taught during the training, such as making an agenda with the patient, pausing, summing up, and checking the patient’s understanding.

According to trainers and DMs, the participants who benefited most from the courses were those with the most open-minded and outgoing personalities and participants who already had good communication skills: “Some types of people find it easier to communicate and learn to communicate. They are more receptive to the course” (DM). Some trainers reflected that nurses seemed to benefit most: “Common to nurses is that they are more motivated [ed. than doctors]”.

Furthermore, the interviewees reported that HCPs employed in the emergency and anaesthesiology departments seemed more sceptical about the benefits of CST. An explanation could be that communication is considered less critical than technical skills within these specialities: “If it had been a new way of plastering, they might have been different” (a trainer’s description of HCP’s attitude in the emergency department).

### 3.5. Lack of Systematic Follow-Up

All trainers were concerned about a lack of systematic follow-up on the CST. A general guideline encouraging the departments to conduct a follow-up annually was part of the concept; however, the execution depended on each department. The trainers were discouraged and frustrated: “Follow-up is missing to bring a lasting change of behaviour”, and “The major weakness is that there is no structured follow-up”. Some trainers suggested bedside training or bringing up communication situations at morning conferences or staff meetings. Interestingly, none of the interviewed DMs discussed the subject; when asked, they responded that they were unaware of the guideline, that follow-up was entirely the trainers’ responsibility, or—displaying a sense of fatigue—that after completing the intervention, communication was not the department’s priority for a while. Only a few of the DMs showed interest and support.

## 4. Discussion

The interviews with the DMs and trainers disclosed the main facilitators and barriers to implementing the 3-day CST programme. Although both the DMs and hospital managers initially agreed on the scope of the implementation, including the mandatory and the top-down approach, this gave rise to some resistance; subsequently, it became necessary to reconsider the definition of “mandatory”. However, the mandatory policy was, at the same time, an essential facilitator of the implementation of the CST. In general, the DMs supported the implementation of the intervention. They were ready to make difficult decisions, reschedule activities, and follow up on the problems that arose, a central prerequisite for a successful intervention [27,47]. The fact that the same CST had been investigated in previous studies in two departments of the hospital [35,36,37] improved the legitimacy of the intervention [47]. It can explain the DMs’ predominantly positive approach and the willingness to implement it at a larger scale [31,48]. Most of the DMs supported this by attending the CST. The fact that HCPs experienced the CST as applicable and valuable was another facilitator. Consequently, staff support is as essential as management support, thus demonstrating the importance of being aware of implementation readiness within different departments when choosing where to initiate large-scale implementations [49]. The relevance and importance of the knowledge provided in this study, despite data being of an earlier date, has renewed topicality given the increased focus on the implementation of CST not only in Denmark but also in Australia, Ireland, Austria [30], the US [50], and Norway [33]. Learnings from the latter were recently published, and the authors mentioned being caught somewhat off-guard regarding resistance and scepticism among participants. Thus, similar implementation barriers seem to exist across settings and countries, and sharing knowledge about CST implementation might be of even greater relevance in the present moment.

Likewise, the train-the-trainer approach is an essential facilitator enabling implementation at an organisational level. Nonetheless, the interviews elucidated that the experienced outcome of the CST depends on the individual trainer’s skills, which corroborates the findings of recent research emphasising the importance of recruiting only HCPs who have demonstrated effective communication skills [30,51]. The trainers are not merely a crucial part of the CST; they also become local ambassadors; consequently, they play a critical role in achieving successful implementation [27].

The barriers emphasised by the majority of DMs were the consumption of staff resources and the challenge of scheduling courses in a busy clinical practice. The DMs emphasised the need for support and supervision during planning and implementation. Consequently, CST programmes may benefit from cooperation between relevant departments, such as the hospital’s communication department, the quality and HR department, and, in this case, the research department. In addition, this type of collaboration could also improve the organisation-wide communication on the purpose of the CST to provide a greater understanding and sense-making for the HCPs, as requested by the DMs and trainers.

Furthermore, as pointed out by the trainers, a plan for follow-up of the CST is needed. Such follow-up can be achieved through a comprehensive collaboration, thereby establishing an organisational process that could contribute to the adaptation and maintenance of the process [47,52].

Other critical barriers mentioned by the interviewees’ concerned seniority, profession, cultural differences, and speciality. The reluctance among senior doctors aligns with an earlier study at Lillebaelt Hospital, where doctors from the orthopaedic department expressed similar opinions after participating in CST [53]. Previous studies have shown that surgeons tend to be mainly disease-oriented [54] and deprioritise interpersonal and personal communication skills and practice-based learning [55]. These findings might partly explain the scepticism observed within the emergency and anaesthesiology departments. Barriers within profession and speciality highlight the need for greater attention to the differences in clinical settings when planning the implementation of a relatively generic intervention.

The interviewees’ reports of a positive change once the HCPs had attended the CST were supported by quantitative studies evaluating this specific CST programme, showing that the HCPs improved their communication in patient consultations after the implementation [44]. Within all professions, communicative self-efficacy and experience of perceived importance improved significantly immediately after completion of the CST. Furthermore, self-efficacy remained high, indicating the positive impact of using the taught skills in daily practice [45].

This study has some limitations. The main limitation is the loss of some audio files, leaving us solely with the initially transcribed citations, causing inconsistencies in the data analysis. Furthermore, for this evaluation, we enrolled only the first 11 departments, equally distributed between surgical, medical, and clinical-service departments, involved in the implementation. This was decided due to the long process of implementing all the departments, as the hospital only had the capacity to conduct the course in a few departments simultaneously. Consequently, some subspecialties were not included in the evaluation. Nevertheless, the results seemed highly consistent across the participating departments, except for the emergency and anaesthesiology departments, in which the HCPs argued for a shorter course. In addition, we reached thematic saturation during the analysis, and it is thus rather unlikely that we would have uncovered further essential aspects if including even more departments.

## 5. Conclusions

This study elucidates barriers and facilitators when implementing CST in an entire hospital. The main barriers proved to be resource requirements, planning, and dealing with reluctance among some HCPs. Notably, most of the scepticism and reluctance faded and even changed to satisfaction after participating in the CST; moreover, most HCPs gave favourable feedback on the training. The fact that the initial resistance among some HCPs vanished in the course of the CST should be considered when planning and studying the implementation of CST programmes—how can the initial reluctance and even resistance be avoided? An essential facilitator was support from management at all levels. In particular, the DMs played a crucial role in successfully implementing the program, and their support to both trainers and staff was of great importance during the process. However, attention is needed regarding adjustment to the specific clinical context, systematic follow-up, and the continuous training of trainers.

When implementing similar programmes, extensive support from managers at all levels and an awareness of the use of resources needed to prepare the process is required. Other activities in the organisation should be prioritised accordingly. Choosing a mandatory approach requires that the implication of ‘mandatory’ is defined and communicated explicitly to the staff. To achieve long-lasting changes in communication practices, a systematic follow-up to support and maintain the transfer of taught skills to clinical practice is essential, as is a follow-up on the competencies of both the trainers and the HCPs.

## Figures and Tables

**Table 1:** Number of interview participants from the included departments.

Department, Location	Number of Interview Participants	
	DM Group Interviews	Trainers Individual Interviews
Anaesthesiology, Kolding	2	none
Anaesthesiology, Vejle	3	2 ^†^
Emergency, Kolding	3	none
Emergency, Vejle	2	1
Internal Medicine, Kolding	none	1 ^†^
Neurology, Kolding	2	1
Orthopaedic Surgery, Kolding	2 ^†^	1
Orthopaedic Surgery, Vejle	2	1 ^†^
Rehabilitation, Kolding & Vejle	2	none
Surgery, Kolding	3 ^†^	none
Vascular Surgery, Kolding	2	3 ^†^

^†^ Only previously transcribed citations were accessible; see Section 2.6. Data analysis for further information.

**Table 2:** Themes and subthemes derived from the thematic analysis.

Themes	Subthemes
Resource consumption	
Obstacles	a. Teaching methods b. Seniority, profession, and cultural differences c. The trainers’ competences d. Sense-making e. Transforming reluctance by doing
Management support	
Efforts and outcomes	
Lack of systematic follow-up	

## Data Availability

The interview data that support the findings of this study are in Danish. Data are available from the corresponding author (M.W.) on reasonable request.
